# Trends and Scientific Production on Isometric Training: A Bibliometric Analysis

**DOI:** 10.3390/sports13050145

**Published:** 2025-05-12

**Authors:** Mario Ríos Riquelme, Ángel Denche-Zamorano, Diana Salas-Gómez, Antonio Castillo-Paredes, Gerson Ferrari, Cecilia Marín-Guajardo, Juan Francisco Loro-Ferrer

**Affiliations:** 1 Escuela de Doctorado de la ULPGC, Universidad de Las Palmas de Gran Canaria, 35001 Las Palmas de Gran Canaria, Spain; mario.rios101@alu.ulpgc.es; 2 Escuela de Ciencias de la Actividad Física, el Deporte y la Salud, Universidad de Santiago de Chile (USACH), Santiago 9170022, Chile; gerson.demoraes@usach.cl (G.F.); cecilia.marin@usach.cl (C.M.-G.); 3 Promoting a Healthy Society Research Group (PHeSO), Faculty of Sport Sciences, University of Extremadura, 10003 Cáceres, Spain; denchezamorano@unex.es (Á.D.-Z.); diana.salas.gom@gmail.com (D.S.-G.); 4 Departamento de Desporto e Saúde, Escola de Saúde e Desenvolvimento Humano, Universidade de Évora, 7004-516 Évora, Portugal; 5 Grupo AFySE, Investigación en Actividad Física y Salud Escolar, Escuela de Pedagogía en Educación Física, Facultad de Educación, Universidad de Las Américas, Santiago 8370040, Chile; 6 Faculty of Health Sciences, Universidad Autónoma de Chile, Providencia 7500912, Chile; 7 Departamento Ciencias Clínicas, Facultad de Ciencias de la Salud, Universidad de Las Palmas de Gran Canaria, 35016 Las Palmas de Gran Canaria, Spain; juanfrancisco.loro@ulpgc.es

**Keywords:** aging, bibliometric analysis, cardiovascular health, isometric training, rehabilitation

## Abstract

Isometric training is a method focused on muscle strengthening without joint movement and has gained attention in recent years due to its applicability in rehabilitation and sports medicine. However, no comprehensive bibliometric analysis focused exclusively on adult populations has been performed. This study aimed to analyze the scientific production related to isometric training in adults; identify prominent authors, journals, and thematic trends; and evaluate the evolution of interest in this field over time. A bibliometric review was performed using the Web of Science Core Collection (SCI-E, SSCI, and ESCI). A specific search strategy was applied to identify articles and reviews focused on isometric training in adult populations. A total of 238 records met the inclusion criteria. Data were analyzed using Excel 2016 and VOSviewer software1.6.20. Bibliometric indicators such as Price’s Law, Bradford’s Law, Lotka’s Law, h-index, and co-occurrence and co-authorship network analysis were applied. The results showed a steady increase in publications in the last decade, highlighting the categories of Sports Science, Physiology, and Cardiovascular. The *Journal of Applied Physiology* was the most frequent source, and Springer Nature was the most prolific publisher. The h-index identified 21 highly cited papers, and Lotka’s Law confirmed the existence of a small group of prolific authors. VOSviewer analysis revealed clear thematic clusters, mainly around blood pressure regulation, rehabilitation, and aging. International collaboration was evident, with the United States, Canada, and the United Kingdom leading the co-authorship networks. Scientific interest in isometric training for adult populations is growing, particularly in relation to cardiovascular health and rehabilitation. Despite this, gaps remain in terms of methodological consistency and standardized protocols. Addressing these issues could improve the applicability and scientific impact of this training modality.

## 1. Introduction

Isometric training is characterized by muscle contraction maintained in a static position, where no joint movement occurs. This type of exercise is effective for improving strength at a specific angle, and it is frequently used in rehabilitation programs, localized strengthening, and in sports that require stability and postural control [1]. Isometric training has been widely studied since its benefits include the ability to generate significant improvements in muscle strength without increasing the amplitude of the joint range [2]. This concept was introduced by Hettinger & Müller in 1953 [3,4,5] and has since been used in a variety of contexts and populations, ranging from sports performance to rehabilitation, being beneficial for both athletes and people with various medical conditions. Its ability to strengthen muscles without requiring joint movement makes it specifically useful in injury recovery, as it is able to minimize stress on joints [6]. In addition, its effectiveness in improving strength in specific positions makes it a valuable option for athletes seeking to improve their performance [1]. This is how its versatility has allowed its usage in various contexts, such as rehabilitation, physical preparation of athletes, special populations, and the general public [7].

A key area of research and one of its most prominent uses has been in the area of health, specifically in its effect on the control of high blood pressure. In this regard, some studies have shown that isometric resistance training (IRT) effectively reduces blood pressure, beyond what can be achieved with aerobic or dynamic resistance training [8,9]. Combining isometric training with inspiratory muscle exercises has also been shown to be an effective strategy for lowering blood pressure in older adults [10,11]. This type of training, in addition to being inexpensive and easy to implement, has demonstrated value as an intervention for hypertension control in various adult populations [12,13] or in older people with reduced mobility [14]. It has also been successfully used in the rehabilitation of patients with coronary artery disease, where it has been shown to improve functional outcomes without significant risks [15]. This approach has even been applied to the treatment of chronic tendinopathies, where isometric training, although not always superior to isotonic exercise, has shown benefits in combination with a progressive loading program [6,16]. Another area in which isometric training has shown benefits is in the treatment of chronic, non-specific neck pain in women, aged 25–53 years, where isometric exercises have been shown not only to significantly improve joint mobility, but also to reduce pain [17,18]. Similarly, in patients with knee osteoarthritis, this type of training has shown positive effects both in decreasing pain and improving joint function (climbing and descending stairs) [19]. In addition, isometric exercises combined with electrostimulation have been effective in improving joint function and reducing inflammation in patients with early-stage knee osteoarthritis [20].

In sports, isometric training has been key to improving dynamic performance in activities such as running, jumping, or cycling, due to its ability to increase strength in specific positions [1,15]. Isometric training has also demonstrated positive effects on muscle stiffness, contributing to improved load capacity and ballistic performance of the limbs [21]. One study suggests that this type of training reduces micromotions in the vertebrae, so it can be a valuable tool in injury prevention [22]. On the other hand, the combined use of isometric training with electromyographic biofeedback (EMGBF) has shown additional improvements in both muscle strength, pain reduction, and increased muscle thickness in patients with knee osteoarthritis [23].

Bibliometric analysis has become increasingly important in business and scientific research, helping to evaluate scientific output, detect trends, and support strategic decisions. In business studies, it reveals key authors, topics, and collaboration networks essential for understanding innovation [24]. Identifying emerging trends in the performance of articles and journals allows researchers to track the evolution of a scientific domain, highlighting which topics are gaining momentum and which publication outlets are most influential. This process is especially useful for recognizing shifts in research priorities, methodological innovations, and thematic consolidation. According to Donthu et al. [24], bibliometric performance analysis enables the discovery of citation classics and high-impact articles that have shaped a field. Furthermore, tracking journal productivity and influence, as proposed by Bradford’s Law [25], helps delineate core publication venues that concentrate the majority of scientific output. These insights are essential for guiding researchers in selecting suitable journals and understanding the competitive landscape of scientific communication. Exploring the intellectual structure of a specific research domain is essential to understanding its theoretical foundations, prevailing schools of thought, and conceptual evolution. Co-citation analysis, keyword co-occurrence, and author co-authorship networks allow researchers to map thematic clusters and reveal how knowledge is interconnected across time. As Small demonstrated, co-citation patterns reflect the intellectual linkages that form the core of scientific paradigms [26]. Similarly, White and Griffith highlighted that intellectual structure analysis provides a systematic approach to identify research fronts and thematic boundaries [27]. These techniques help scholars contextualize new research within an established knowledge framework and uncover gaps that warrant further exploration.

When reviewing the scientific literature on this topic, numerous reviews of different types were found, including systematic reviews and meta-analyses, narrative reviews, and umbrella reviews [2,11,28,29,30,31]. However, no bibliometric analysis was found that performed an analysis of the current state of scientific production related to isometric training in adults and older adults. Therefore, this study is the first bibliometric analysis based on the traditional laws of bibliometrics in which the main objectives were as follows: (1) to verify whether the scientific production in this subject is in a phase of exponential growth; (2) to highlight the most cited papers; (3) to identify the most productive and prominent authors in this object of study; (4) to point out the core journals where the authors publish the most; (5) to discover the keywords and topics most addressed by authors in the studies of this object. The starting hypotheses were (1) scientific production on this object of study is in a phase of exponential growth; (2) there is a small group of authors and journals that accumulate most of the scientific papers on this topic according to the traditional laws of bibliometrics; (3) there is a small group of authors and journals that are the most productive in this object of study; (4) there is a small group of authors and journals that accumulate most of the scientific papers on this topic according to the traditional laws of bibliometrics.

## 2. Materials and Methods

### Type of Study

The type of study employed was a bibliometric analysis. The methodology applied was based on traditional bibliometric laws. A search was carried out from articles published in journals indexed in the main collection of Web of Science [32]. The following databases were selected: the Science Citation Index Expanded (SCI-E), the Social Science Citation Index (SSCI), and the Emerging Sources Citation Index (ESCI).

A systematic search was carried out through the advanced search option. The following search vector was used to obtain the set of documents: (Ti = (((“isometric training” or “isometric exercise” or “isometric resistance training”) AND (“adult*” OR “aging” OR “elder*” OR “senior*” OR “aged”))) OR AB = ((“isometric training” or “isometric exercise” or “isometric resistance training”) AND (“adult*” OR “aging” OR “elder*” OR “senior*” OR “aged”))). The search vector included the tags TI (title searches), AB (abstract searches), and the asterisk ‘*’ (in this case, search for compound terms containing the word “elder” or “adult” or “senior”). In addition, words in quotation marks were used to search for that word in isolation or, in the case of compound words, to search for terms that appeared together. The following inclusion criteria were established: (a) type of studies: Articles and Reviews; (b) meeting the search criteria; (c) studies conducted in humans. No limitations, such as years of publication or language exclusions, were applied.

Researchers A.D.-Z. and D.S.-G. carried out the search on 27 September 2024 to ensure consistency in the set of articles obtained. The documents were extracted in both plain text and .xls (Microsoft Excel) formats. Co-authors A.D.-Z. and D.S.-G. reviewed all papers to ensure that they met the inclusion criteria. Any studies that did not meet the criteria were eliminated.

The following analyses were carried out on the resulting set of documents: To check whether the subject is currently in an exponential growth phase, the Price/Dobrov exponential growth law was applied, analyzing the years of publication and checking the trend followed by the annual publications on the basis of the adjusted coefficient of determination (R^2^) [33,34]. Exponential growth of an object of study indicates a growing interest among researchers and a large critical mass for its development. A graph was made with Excel to represent the current trend of the publications. Subsequently, a descriptive analysis of the WoS subject categories with the highest number of publications was carried out.

The Hirsch Index (h-index) was used to identify the most cited documents. To make this determination, the documents were ordered in decreasing order according to the number of WoS citations received, identifying as the most cited documents the h document with h or more citations [35,36,37]. A graph of the distribution of documents and citations was made to establish the cut-off point with the h-index. In addition, measures of central tendency and dispersion (mean, median, interquartile range, and outliers) were calculated for the citations of the documents found. These measures were presented graphically in a box plot.

Subsequently, the most specialized journals in the field were identified using Bradford’s law [38,39,40]. By applying this law, we determined the nucleus of journals, which comprised the first tercile of documents. A descriptive analysis was then conducted on these core journals, including details such as journal name, publisher, number of documents, citations, normalized citations (citations per document), Journal Impact Factor (JIF), quartile, and the percentage of Gold Open Access.

For the analysis of authors, firstly, authors’ names were standardized (eliminating duplicates), and Lotka’s law was utilized to estimate the number of prolific authors [41,42,43,44]. This number was calculated as the square root of the total number of authors. To verify the correct application of Lotka’s law, a discrete count of the number of papers per co-author and the number of co-authors per level of production was carried out. Additionally, co-authorship graphs were generated using VOS viewer software1.6.20 with the identified authors [45,46]. Prolific co-authors with one or more documents among the most cited documents were identified as prominent co-authors [40,47,48,49]. A descriptive analysis of the prominent co-authors was carried out, showing their names, countries, number of articles, number of citations, number of most cited articles, and color of the production clusters with which they were associated. In addition, a descriptive analysis of co-authorship by region/country was carried out to identify those with the highest number of articles and citations. Co-authorship graphs were generated with VOS viewer software to visualize international production networks [50].

Co-authored countries were analyzed, checking the number of papers and citations per country. A graph was made in Microsoft Excel to show the co-authoring countries as a function of the number of total citations for each country. A country co-authorship analysis was performed in VOS viewer to show the global production network, and the size of the nodes was presented as a function of the number of papers, and the links indicated the connections between co-authoring countries.

Finally, to identify the author keywords and Keywords Plus^®^ with the highest co-occurrence in the set of documents, Zipf’s law was applied [40,51,52]. Regarding Keywords and Keywords Plus^®^, the latter is related to the frequent use of words or phrases associated with article titles that are not necessarily available in the keywords. Keywords Plus^®^ is based on an exclusive Clarivate algorithm. Co-occurrence analyses were conducted in VOS viewer using the most frequently co-occurring keywords and Keywords Plus^®^ to identify potential thematic clusters and determine the average publication year for each term [50]. Subsequently, a graph showing the frequency of use of the number of keywords and Keywords Plus^®^ identified after the application of Zipf’s law was made. All analyses and graphs were carried out with the VOS viewer software [45] and Microsoft Excel 2016 (Supplementary Table S1).

## 3. Results

### 3.1. Network Metrics

A total of 269 documents were found, although only 238 met the inclusion and exclusion criteria. Fifteen papers were excluded because they were not articles or reviews, five papers because they were animal research, three papers because they were research on children or adolescents, and one paper because it was research on in vitro muscle cells (Figure S1, Flow chart).

#### 3.1.1. Annual Trend of Publications

**Figure 1:**
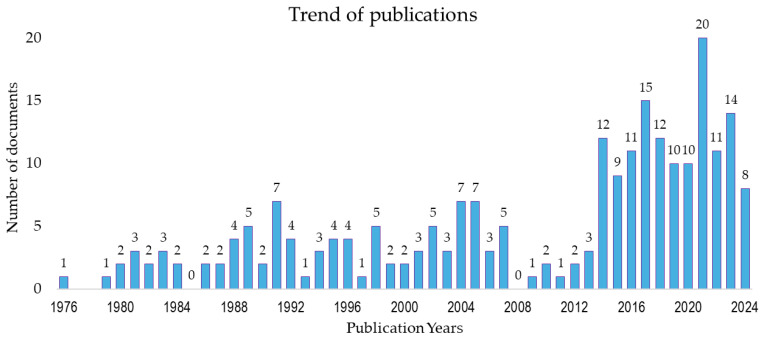
Trend of annual publications.

The first paper on the subject was found to have been published in 1976 [53], although the trend of publications has evolved irregularly up to the present day. After the publication of the first paper, a new paper was not found until 1979 [54]. Since then, there have been other periods of continuous publications, followed by years without publication: 1979–1984, 1986–2007, and 2009–2024 (until 27 September 2024). By applying the Price/Dobrov law, it was found that annual publications in this last period were not in an exponential growth phase (R^2^ = 50%). However, the number of publications on the subject has doubled in the last 10 years (120 papers between 2015 and 2024), surpassing the number of papers published in the previous 37 years (116 papers between 1976 and 2014). Figure 1 shows the trend of annual publications.

#### 3.1.2. Thematic Categories

A wide variety of thematic categories were found in the review documents. Thus, the documents were related to 62 WoS thematic categories with a range of documents between 1 and 55. The thematic category to which most documents were related was Sport Science, with 55 documents, with the *Journal of Applied Physiology* (8 documents) and Springer Nature (13 documents) being the journal and publisher with the highest number of documents contributing to the category, respectively. Table 1 shows the five thematic categories with the highest number of documents: Physiology (42 papers), Peripheral Vascular Disease (32 papers), Cardiac Vascular System (30 papers), and Medicine General Internal (21 papers), as well as the journals and publishers with the most papers in each. Other outstanding categories were Rehabilitation (15 documents), Ophthalmology (13 documents), Geriatrics Gerontology (12 documents), Clinical Neurology (10 documents), and Pharmacology Pharmacy (9 documents).

#### 3.1.3. Most Cited Papers

The papers presented a range of citations from 0 to 808, with a mean number of citations of 31 and a median of 12 (IQR = 31; Q1 = 3, Q3 = 34) (Figure S2: Box plot).

**Figure 2:**
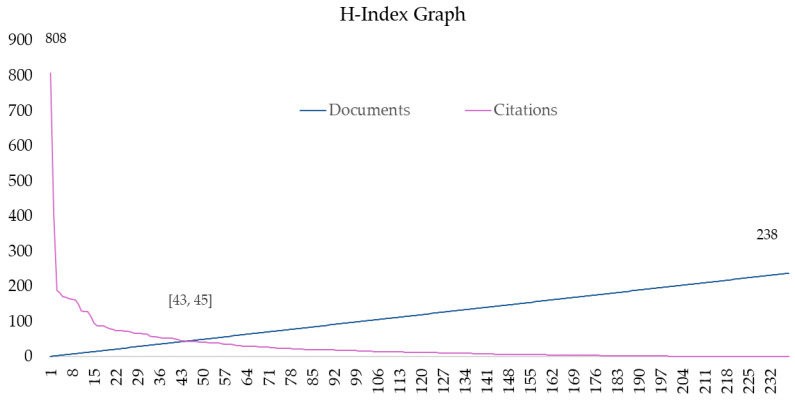
H-Index graph.

Applying the h-Index, 43 documents with 45 or more citations stood out (Figure 2). All these papers were found to be above the median citation rate of the dataset and above the third quartile.

Table S1 shows the 43 most cited papers. Notably, a review published by Cornelissen et al. [25] stood out far above all by accumulating 808 citations, doubling or quadrupling the next most cited papers, which presented 407 [55], 188 [30], 185 [8], or 177 [56] citations. The top four most cited papers were reviews.

#### 3.1.4. Prolific and Prominent Authors

**Figure 3:**
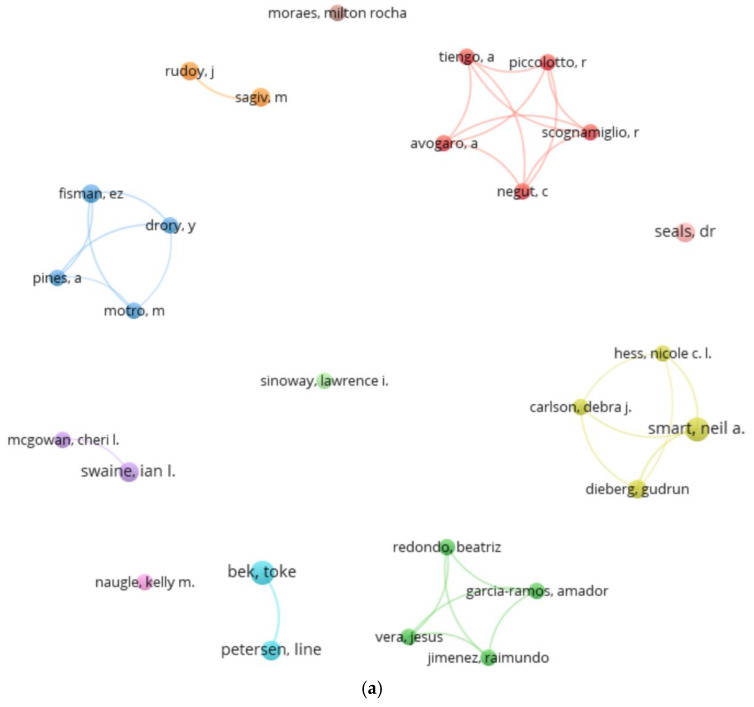

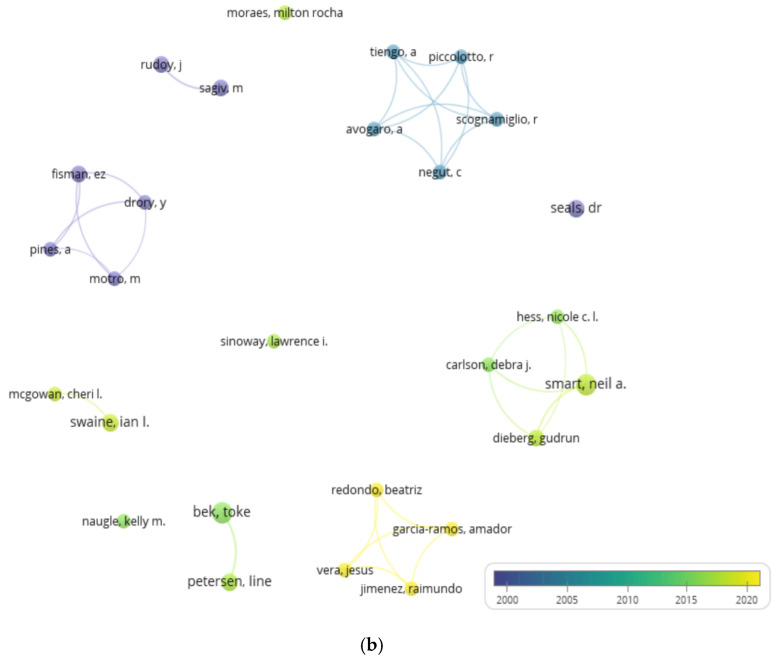
(**a**) Prolific co-authors graph. (**b**) Prolific co-authors graph: average publication years.

A total of 1041 co-authors published papers. These co-authors presented a publication range between 1 and 8 papers. Applying Lotka’s law, it was estimated that prolific co-authors should be equal to or less than 32 (the square root of 1041). We found 27 co-authors with three or more papers, considering these as prolific co-authors. In contrast, a high volume of co-authors with 1 (934 co-authors) or 2 papers (80 co-authors) was found. Figure 3 shows the 27 prolific co-authors and the production network they form when performing a co-authorship analysis.

According to the level of production, these authors included Toke Bek and Neil A. Smart (8 papers), followed by L. Petersen, D. R. Seals, I., and I. L. Swaine (5 papers). Among the prolific co-authors were four collaborative groups: the Red Cluster (Avogaro, Tiengo, Negut, Scognamiglio, and Piccolotto), Yellow Cluster (Smart, Dieberg, Carlos, and Hess), Green Cluster (Vera, Garcia-Ramos, Jimenez, and Redondo), and Blue Cluster (Motro, Drory, Pines, and Fisman). These two clusters had the most recent (2022) and the oldest (1988) mean year of publication, respectively (Figure 3b).

When checking for prolific co-authors who submitted at least one paper among the most cited, the number of prolific co-authors was reduced to 9 prominent co-authors. Thus, the prominent co-authors were D. R. Seals (5 papers, 476 citations and 5 most cited papers) and N. A. Smart (8 papers, 1276 citations and 4 most cited papers). In addition to other authors such as G. Dieberg, D. Carlson, N. Hess, T. Bek, k. Naugle, I.A. Swaine, and C.L. Mcgowan, with between 1 and 5 papers among the most cited (Table 2).

#### 3.1.5. Countries/Regions

**Figure 4:**
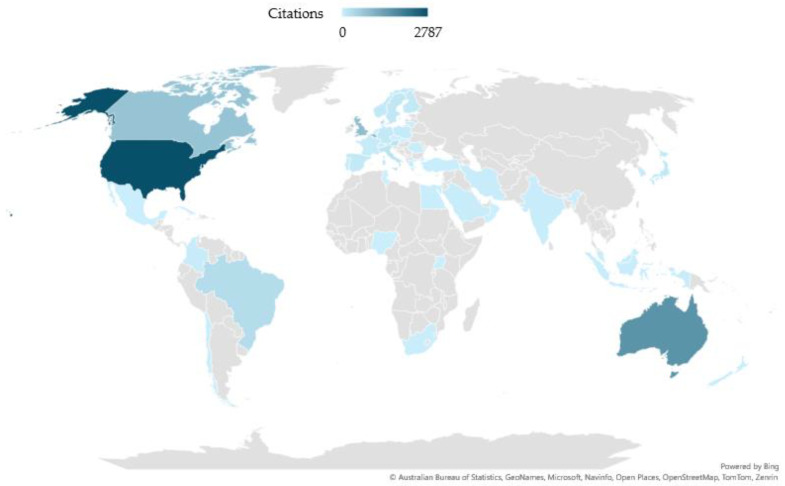
World map of countries in co-authorship. Scores based on the number of citations.

**Figure 5:**
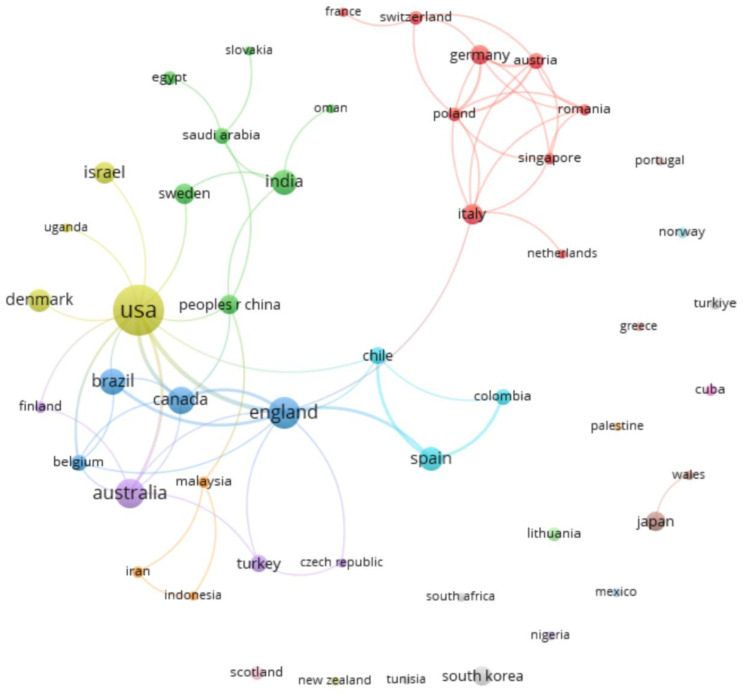
Countries/regions co-authorship graph.

When co-authorships were analyzed by country, a total of 50 countries/regions were found with a range of publications between 1 and 66 documents. The range of citations was between 0 and 2787 citations. The United States of America (USA) was the country with the highest number of papers and citations (66 papers and 2787 citations). Specifically, the number of papers tripled or quadrupled in the following countries/regions: England (21 papers and 746 citations), Australia (18 papers and 1588 citations), Canada (13 papers and 742 citations), or Brazil (13 papers and 322 citations). Figure 4 shows the world map of co-authoring countries according to the number of citations, while Figure 5 shows the co-authoring countries and the production networks formed between them.

In the co-authorship analysis, two large collaborative networks were found, formed by 9 (Red Cluster) and 7 (Green Cluster) countries/regions (Figure 5). The most numerous cluster (Red Cluster) was formed around European countries such as Italy, Germany, Poland, and Austria, among others. The second largest cluster (Green Cluster) was formed around countries such as India, China, Sweden, and Saudi Arabia. The USA, despite being the country with the largest number of documents and the largest number of international collaborations (12 links), was in one of the smallest clusters, including countries such as Denmark, Uganda, and Israel (Yellow Cluster).

#### 3.1.6. Nucleus of Journal

The papers were published in a total of 238 journals with a publication range between 1 and 8 papers. It was found that the papers did not follow Bradford’s distribution in the journals. A core of 19 journals was found to publish 32% of the papers (75 papers), with a publication range between 3 and 8 papers. However, a high peripheral dispersion of publications was found, and the rest of the documents were not distributed in two zones by terciles (Zone 1 and Zone 2) but were distributed in a single zone (Zone 1). Zone 1 was formed by 143 journals with a publication range of 1 or 2 documents on the subject, accumulating a total of 163 documents (68% of the total number of documents). Table 3 shows the 19 journals that made up the core group of journals.

Among these journals, the *Journal of Applied Physiology* (8 papers and 468 citations), *European Journal of Applied Physiology* (7 papers and 166 citations), *American Journal of Physiology-Heart and Circulatory Physiology* (6 papers and 172 citations), and *Journal of Hypertension* (5 papers and 167 citations) stood out. By number of citations, among these journals, the *Journal of Pain* (3 papers and 470 citations) stood out.

#### 3.1.7. Keywords and Keywords Plus

**Figure 6:**
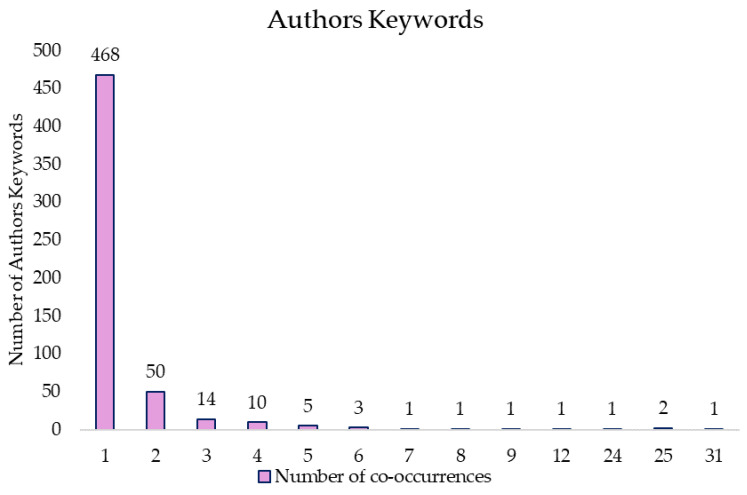
Authors keywords distribution.

The co-authors used a total of 558 keywords in the document set. Figure 6 shows the distribution of keywords according to the number of co-occurrences of keywords.

As can be seen in Figure 6, more than 90% of the keywords had co-occurrences of less than two documents. On the contrary, the words with the highest co-occurrence appeared between 25 and 31 documents. Applying Zipf’s law, it was estimated that the 24 keywords (square root of 558 total keywords) with the highest co-occurrence should be considered the most relevant to the co-authors. However, 26 keywords with 4 or more co-occurrences were found, considering these as the most relevant words for the co-authors. These words and their level of co-occurrence are shown in Figure S3: Iso-metric Exercise (31 documents), Blood Pressure and Hypertension (25 documents), and Exercise (24 documents).

**Figure 7:**
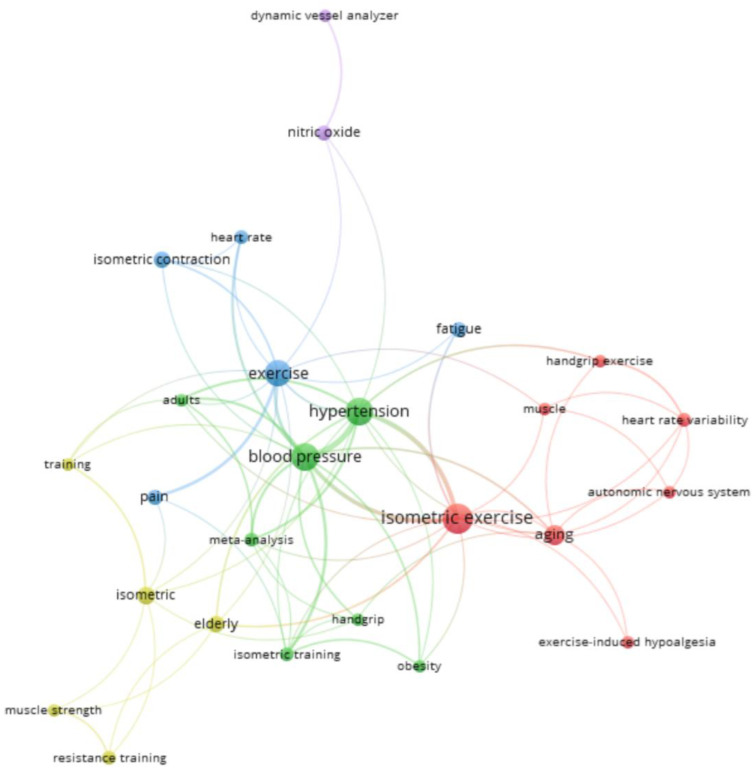
Authors keywords co-occurrence graph.

In the co-occurrence analysis with the 26 most used keywords, five thematic clusters were found. The two clusters with the highest number of keywords were the Red Cluster (5 keywords: Isometric Exercise, Aging, Exercise-Induced Hypoalgesia, Autonomic Nervous System, Heart Rate Variability, among others) and the Green Cluster (Blood Pressure, Hypertension, Isometric Training, Obesity, among others) (Figure 7). Figure S4 shows these words according to the mean year of their publications. The terms with the most recent mean year of publication were Isometric Training (2022), Training (2020), and Meta Analysis (2020).

**Figure 8:**
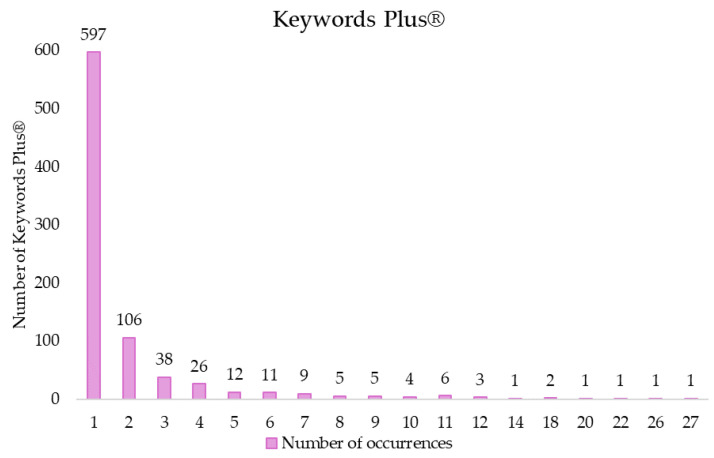
Keywords Plus^®^ distribution.

As for Keywords Plus^®^, 829 terms were found with a co-occurrence between 1 and 27. Figure 8 shows the distribution of these words according to the number of co-occurrences.

By applying Zipf’s law to these words, it was estimated that the most prominent should be the 29 with the highest co-occurrence. Thirty terms were found with 8 or more co-occurrences, these being the most relevant in this field of study. Blood-Pressure (27 documents), Exercise (26 documents), Responses (22 documents), Hypertension (20 documents), together with Physical Activity and Skeletal-Muscle (18 documents), were the most frequent terms (Figure S5).

**Figure 9:**
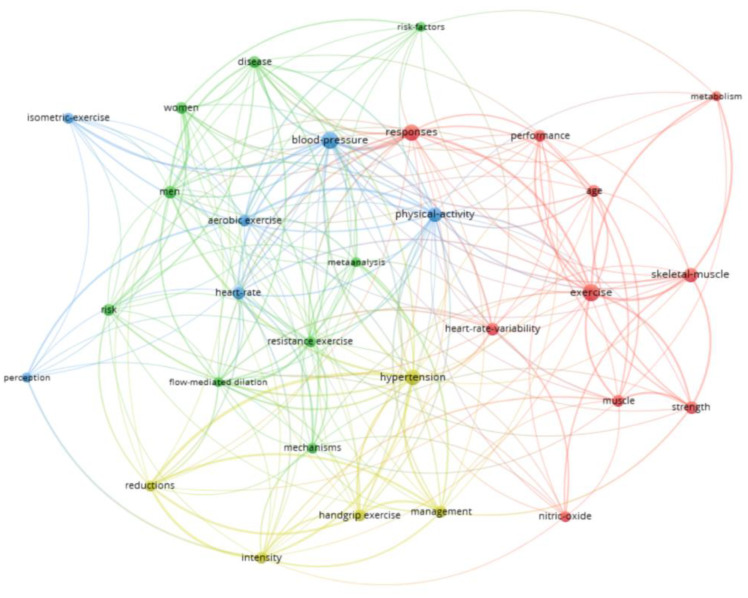
Keywords Plus^®^ co-occurrence graph.

In the co-occurrence analysis, four large thematic clusters were found. The two most numerous had 9 (Green Cluster: Resistance Exercise, Disease, Men, Women, and Risk, among others) and 10 terms (Red Cluster: Exercise, Performance, Nitric-Oxide, Muscle, and Responses, among others). Figure 9 shows the co-occurrence graph of these terms, while Figure S6 shows these same terms as a function of the mean year of their papers.

## 4. Discussion

To the authors’ knowledge, this study is the first bibliometric analysis of scientific production related to isometric training. This research analyzed 238 papers published in journals indexed in three databases, SCI-E, SSCI, and ESCI, included in the WoS main collection. The general objective was to know the current state of scientific production related to this object of study, for which the traditional laws of bibliometrics were applied in order to know the current trend of its scientific production, identify and highlight the most relevant and cited documents, the most productive and prominent authors, journals that made up the core and author keywords and the most used Plus@ keywords.

### 4.1. Annual Growth in Publications

This bibliometric analysis on isometric training provides a global overview of the evolution and current state of scientific production in this field. This analysis reveals a steady, although not exponential, growth in isometric training research in recent years. Although there has been a significant increase in the last decade, with 120 papers published between the years 2015 and 2024, the growth is irregular in previous periods (1979–1980, 1986–2007), suggesting fluctuations in scientific interest in this subject. This significant increase in scientific production over the last decade reflects an increased awareness of the potential benefits of isometric training, both in terms of physical performance and its application in the rehabilitation of populations with various pathologies such as the elderly, hypertensive patients or patients with obesity, as well as its analgesic and pain control effects in people with neck pain or osteoarthritis [8,10,57,58,59].

### 4.2. Categories and Prolific Researchers

An interesting finding of the present analysis is related to the thematic categories identified, where the dominance of the area of “Sport Science” with 55 papers demonstrates that isometric training remains an important focus within sport science. However, it is the high thematic dispersion in the publications that reflects the versatility of isometric training. Although areas such as sport science and physiology, where a greater number of papers are found, there are studies ranging from neuromuscular rehabilitation to cardiac rehabilitation [15,20]. This suggests that isometric training is applicable to a wide range of disciplines, which could explain the dispersion of publications in specialized journals from different areas and reinforces the idea of its applicability in a wide range of populations, from elite athletes to people with reduced mobility or severe medical conditions.

The authors with the largest number of publications on this topic focus on the study of the benefits of isometric training for cardiovascular health, and it is noteworthy that the first four most cited papers according to the h index are reviews, which indicates that reviews and meta-analyses on the effects of isometric training have been of popular interest to the scientific community; among them we can highlight the work of Cornelissen et al. [29], one of the most cited. This study reports that the type of exercise is more effective than other modalities (resistance training, dynamic resistance, and combined training) in lowering blood pressure, especially in older adults. This finding has motivated a large amount of subsequent research, consolidating the use of isometric training in the prevention and treatment of cardiovascular diseases [60]. Similarly, its applicability in the rehabilitation of musculoskeletal injuries, such as osteoarthritis [19] and tendinopathies [61], has been an area of growing interest.

In addition to the thematic dispersion and with respect to the most productive authors, the analysis of co-authorship networks shows a strong collaboration between researchers from countries such as the United States, Canada, and Australia. These countries not only lead in scientific production but also stand out in terms of impact on the scientific community, as evidenced by the high number of citations received by authors from these regions [16,23], which in turn is consistent with the globalization of scientific research and the growing importance of international collaborations in the advancement of knowledge. This could also be related to the availability of resources and funding for research in these countries, as well as the existence of well-established collaborative networks in the field of health and sport.

### 4.3. Most Used Keywords

The analysis of the keywords and their co-occurrences revealed two main thematic clusters, one consisting of terms such as Isometric Exercise, Aging, Exercise-Induced Hypoalgesia, Autonomic Nervous System, Heart Rate Variability, which seems to focus on the effects of isometric exercise on physiology, especially in relation to aging and autonomic regulation of the organism. The other is composed of terms such as Blood Pressure, Hypertension, Isometric Training, and Obesity, which seem to focus on the effects of isometric training on cardiovascular health, especially blood pressure and hypertension, as well as their relationship to diseases such as obesity. Both thematic clusters reflect the growing interest in investigating the effects of isometric training on cardiovascular health, as observed in recent studies [11].

Another aspect to highlight is the increasing inclusion of new technologies, such as electromyographic biofeedback, suggesting a diversification of research interests, which could in turn indicate that the research field is maturing, and that future research could be more related to emerging areas such as the application of isometric training in injury prevention or integrating it with technologies. Electromyography allows for more accurate monitoring of responses during isometric contractions, which facilitates the customization of training protocols and their optimization for different populations [23]. Despite the importance of these advances, it is noted that most studies still focus on traditional clinical applications, such as injury rehabilitation or blood pressure improvement, suggesting that there is great untapped potential in the use of isometric training in other areas.

Even with the progress in research on isometric training, the fact that an exponential growth in scientific production has not been observed suggests the existence of barriers or limitations that hinder its expansion. One possible explanation may be the lack of standardization of isometric training protocols, which makes comparison between studies difficult and limits the generalization of results. In addition, the traditional focus on its clinical use could be limiting the exploration of new applications in the sports setting and in young and healthy populations.

### 4.4. Strengths and Limitations

Like all research, this work is not without strengths and limitations according to its methodology [62,63]. Regarding strengths, among them we find that this bibliometric analisys is the first to comprehensively address the scientific production related to isometric training in adults and older adults/older people, which provides a solid foundation for future research and practical applications, in addition to identifying trends and recognizing gaps in the literature and especially emerging areas that could guide the design of more effective interventions. Covering a broad time range (1976–2024), the analysis includes both pioneering studies and current trends, which provide a comprehensive view of the field. In addition, the methodology is robust and based on traditional laws, such as Price’s law for assessing the growth of scientific production and Bradford’s law for identifying the most relevant journals in the field. These techniques provide a solid quantitative basis that not only facilitates the identification of trends but also the relevance and impact of the most influential authors [64]. Finally, the study allows us to identify the wide range of disciplines where isometric training has varied applications, such as sports medicine, physiology, and rehabilitation. This multidisciplinary perspective not only highlights the versatility of isometric training but also opens the door to future collaborations between different research fields.

About the limitations, this study is based on databases indexed in the WoS core collection, which is recognized for its prestige and widely used by the scientific community. However, by opting for this source, a selection bias is introduced by excluding research from journals that are not indexed. This decision was made in order to ensure the quality of the documents analyzed, guaranteeing that all were peer-reviewed and met the rigorous WoS indexing standards. However, this choice also presents certain limitations, as it does not include high-impact papers published in journals indexed in other databases.

### 4.5. Suggestions and General Practices and Applications for Future Lines of Research

From the results of this bibliometric analysis, several key recommendations can be proposed.

- First, it is essential to establish standardized Web of Science (WoS) categories and select journals that fit the scope of this specific area of training, in order to improve the coherence, visibility, and comparability of studies in future research.
- Second, it is essential to promote international consensus among researchers to consolidate the development of the area of study and standardize methodological and conceptual approaches related to this topic.
- Finally, special attention should be paid to the careful selection and consistent use of keywords.

For example, our analysis showed that the term “isometric” was used infrequently, which may result in the omission of relevant studies and consequently may limit comprehensive development in this field of research.

## 5. Conclusions

The present bibliometric analysis, the first of its kind, has revealed a growing interest in isometric training, especially in the last decade, both in the sports and clinical fields. Despite the irregularity in the growth of scientific production, the field has shown sig-indicant advances, especially in areas such as blood pressure control, rehabilitation of tendinopathies and treatment of the and treatment of osteoarthritis, which highlights the potential of isometric training to improve the health of diverse populations particularly in older adults or people with reduced mobility [8,15].

Analysis of co-authorship networks and leading research countries suggests that the United States, Australia, and Canada are the main centers of scientific production in this field, with highly cited authors laying the groundwork for new research. The increasing adoption of new technologies, such as electromyography and biofeedback, underscores the potential for improving the customization and efficacy of isometric training programs [23]. However, this analysis has also highlighted several limitations that hold back the expansion and consolidation of research. The lack of standardization in intervention protocols makes it difficult to compare results between studies, which limits the generalization of findings and the implementation of optimized programs. Furthermore, despite growing interest, the field has not yet experienced exponential growth, suggesting the need for further research efforts in emerging areas and non-traditional applications of isometric training.

This study provides a solid foundation for future research by highlighting both the advances achieved and the opportunities for expansion in the clinical and sports settings. Overcoming methodological barriers and exploring new applications will be key to consolidating the impact of isometric training on health and physical performance in diverse populations in order to contribute to current trends in scientific production.

Although isometric training has gained recognition for its multiple applications and versatility in health and sport, it still faces major challenges that limit its expansion. The thematic dispersion and lack of steady growth suggest the need for more research in emerging areas and the creation and optimization of protocols for different populations and targets. Overcoming these barriers will allow further consolidation of isometric training in health, sport, and other key population contexts.

Finally, it is relevant to note that, although isometric training has proven to be an effective intervention for the treatment of certain pathologies, there is still a gap in the understanding of the physiological mechanisms underlying its effects. For example, it is still unclear how the duration, intensity and frequency of isometric contractions affect long-term cardiovascular health or the prevention of musculoskeletal injuries, which is an area in need of further research [10,15].

## Figures and Tables

**Table 1:** The top five thematic categories in the Web of Science, according to the number of documents in which publications are indexed.

WoS Categories	Docs.	Journals	Docs.	Publishers	Docs.
Sport Science	55	*Journal of applicated Physiology*	8	Springer Nature	13
Physiology	42	*Journal of applicated Physiology*	8	American Physiological Society	15
Peripheral Vascular Disease	32	*American journal of physiology-heart and circulatory physiology*	16	Lippincott Williams & Wilkins	10
Cardiac Vascular system	30	*Journal of the American heart association*	6	Elsevier	10
Medicine General Internal	21	*Pain Medicine*	4	Oxford University Press	4

Docs: Documents.

**Table 2:** Prominent co-authors.

Author’s Name	Country	Cluster	Doc.	Cit.	Most Cited Papers
Seals, D.R.	USA	n.a.	5	476	5
Smart, N.A.	Australia	Yellow	8	1276	4
Dieberg, G.	Australia	Yellow	4	391	2
Carlson, D.J.	Australia	Yellow	3	384	2
Hess, N.C.L.	Australia	Yellow	3	363	2
Naugle, K.M.	USA	n.a.	3	469	1
Swaine, I.L.	England	Purple	5	155	1
Mcgowan, C.L.	Canada	Purple	3	129	1
Bek, T.	Denmark	Sky Blue	8	122	1

Doc.: Number of documents; Cit.: Citations; USA: United States of America; n.a.: Not applicable.

**Table 3:** Nucleus of journals.

Publication Titles (Publishers)	Doc.	Cit.	JIF	Quartile	% O.A.
J. of Applied Physiology (American Physiological Soc)	8	468	3.3	Q1	8.1%
European J. of Applied Physiology (Springer)	7	166	2.8	Q1	38.3%
American J. of Physiology-Heart and Circulatory Physiology (American Physiological Soc)	6	172	4.1	Q1	7.1%
J. of Hypertension (Lippincott Williams & Wilkins)	5	167	3.3	Q1	22.0%
American J. of Cardiology (Elsevier)	4	168	2.3	Q2	14.6%
European J. of Applied Physiology and Occupational Physiology (Springer)	4	126	n.a.	n.a.	n.a.
Investigative Ophthalmology & Visual Science (Assoc Research Vision Ophthalmology Inc.)	4	113	5.0	Q1	99.1%
Pain Medicine (Oxford Univ Press)	4	71	2.9	Q1	18.5%
J. of Pain (Churchill Livingstone)	3	470	4.0	Q1	66.4%
American J. of Hypertension (Oxford Univ Press)	3	83	3.2	Q2	21.2%
Hypertension (Lippincott Williams & Wilkins)	3	69	6.9	Q1	18.0%
Gerontology (Karger)	3	68	3.1	Q3	31.2%
Clinical Interventions in Aging (Dove Medial Press LTD)	3	63	3.5	Q2	98.2%
Medicine & Science in Sports & Exercise (Lippincott Williams & Wilkins)	3	43	4.1	Q1	15.6%
Graefes Archive for Clinical and Experimental Ophthalmology (Springer)	3	31	2.4	Q2	29.2%
Scandinavian J. of Medicine & Science in Sports (Willey)	3	30	3.5	Q1	46.0%
J. of Physical Therapy Science (Soc Physical Therapy Science)	3	25	n.a.	n.a.	n.a.
Isokinetics and Exercise Science (IOS Press)	3	14	0.6	Q4	5.6%
J. of Clinical and Diagnostic Research (Premchand Sha ntidevi Research Foundation)	3	8	0.2	Q4	98.5%

Doc.: Number of documents; Cit.: Citations; JIF: Journal Impact Factor; Quartile: JIF Quartile; % O.A.: Percentage of Open Access; J.: Journal.

## Supplementary Materials

The following supporting information can be downloaded at https://www.mdpi.com/article/10.3390/sports13050145/s1. Figure S1: Flowchart; Figure S2: Box Plot Isometric; Figure S3: Authors Keyword; Figure S4: AK as 5_0 AVG PUB YEAR; Figure S5: Keywords Plus; Figure S6: KP as 5_0 AVG PUB YEARS; Table S1: Guidelines, guiding questions and good practices for bibliometric analysis.
