# Supplementary material for: Trends and Scientific Production on Isometric Training: A Bibliometric Analysis
Source: Sports (Basel). 2025 May 12;13(5):145. doi: 10.3390/sports13050145 (PMC12115907; doi:10.3390/sports13050145)
Supplement: Supplementary file 1 [file sports-13-00145-s001.zip › sports-3577893-supplementary.pdf]

Supplementary Table S1. Guidelines, guiding questions and good practices for bibliometric analysis

Analysis stage	Key questions for researchers	Best Practices / Recommendations
1. Definition of the research objective	What is the specific topic or phenomenon to be mapped, and is it delimitable from a bibliometric approach?	Clearly define the object of study (e.g., population, type of intervention, time period). Use precise keywords.
2. Selection of database(s)	Is the selected database suitable for the subject area?	Use recognized databases (Web of Science). Avoid mixing sources without methodological justification.
3. Search strategy and vector	Are the search terms specific but comprehensive? Were the Boolean operators used correctly?	Include synonyms, variants and wildcards (*). Use search fields such as title (TI) and abstract (AB).
4. Inclusion and exclusion criteria	Are the criteria clear, transparent and reproducible?	Define document type, population, language, among others. Manually verify that the results meet the criteria.
5. Data cleaning and preparation	Have the names of authors and journals been standardized to avoid duplication?	Standardize names, institutions and sources. Use Excel, OpenRefine or other data cleaning software.
6. Analysis of scientific production	What is the trend of publications over time, is it linear or exponential?	Apply Price's Law. Graph the annual trend. Calculate the coefficient of determination (R^2).
7. Citation analysis	Which documents have the greatest impact? What is the h-index of the set?	Sort by number of citations. Calculate the h-index. Represent the distribution visually.
8. Journal analysis	Which are the most relevant journals, and is production concentrated in a few?	Apply Bradford's Law. Identify core journals. Include impact factors and open access.
9. Analysis of authorship and co-authorship	Who are the most prolific and influential authors? Are there collaboration networks?	Apply Lotka's Law. Generate co-authorship maps with VOSviewer.
10. Thematic mapping by keywords	What are the most frequent and emerging issues?	Analyze Keywords and Keywords Plus®. Apply Zipf's Law. Identify thematic clusters with VOSviewer.
11. Geographic analysis of collaboration	Which countries have the largest production and are there international collaboration networks?	Analyze co-authorship by country. Visualize nodes and links in network maps.

12. Interpretation and projection	What gaps or emerging lines are identified? What does the structure reveal about the maturity of the field?	Compare clusters with thematic categories. Identify saturated or little explored areas. Propose future lines.
