# Supplementary figures and images for: Trends and Scientific Production on Isometric Training: A Bibliometric Analysis
Source: Sports (Basel). 2025 May 12;13(5):145. doi: 10.3390/sports13050145 (PMC12115907; doi:10.3390/sports13050145)

### Identification of studies via databases and registers

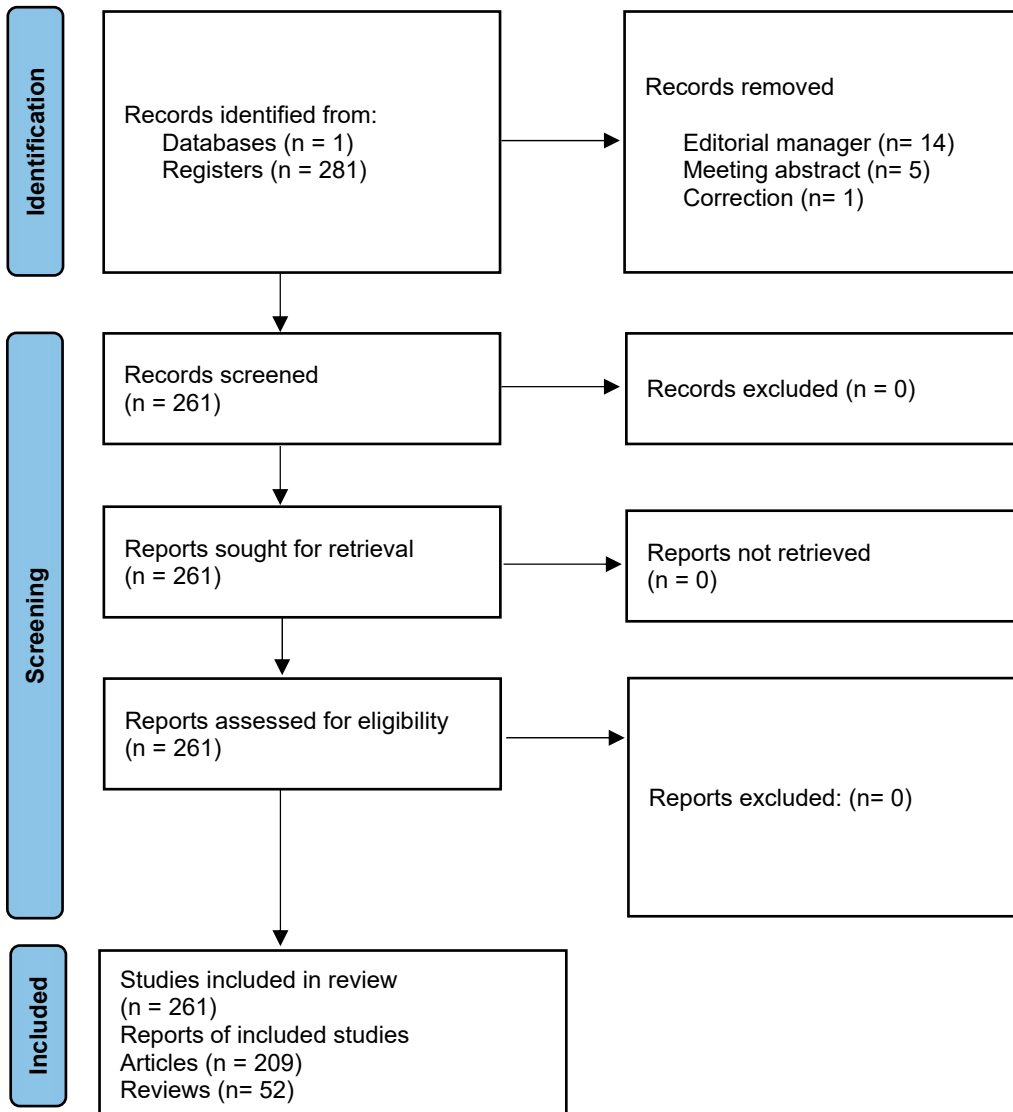

Supplement: Supplementary file 1 [file sports-13-00145-s001.zip › Figure S1. Flowchart.pdf]

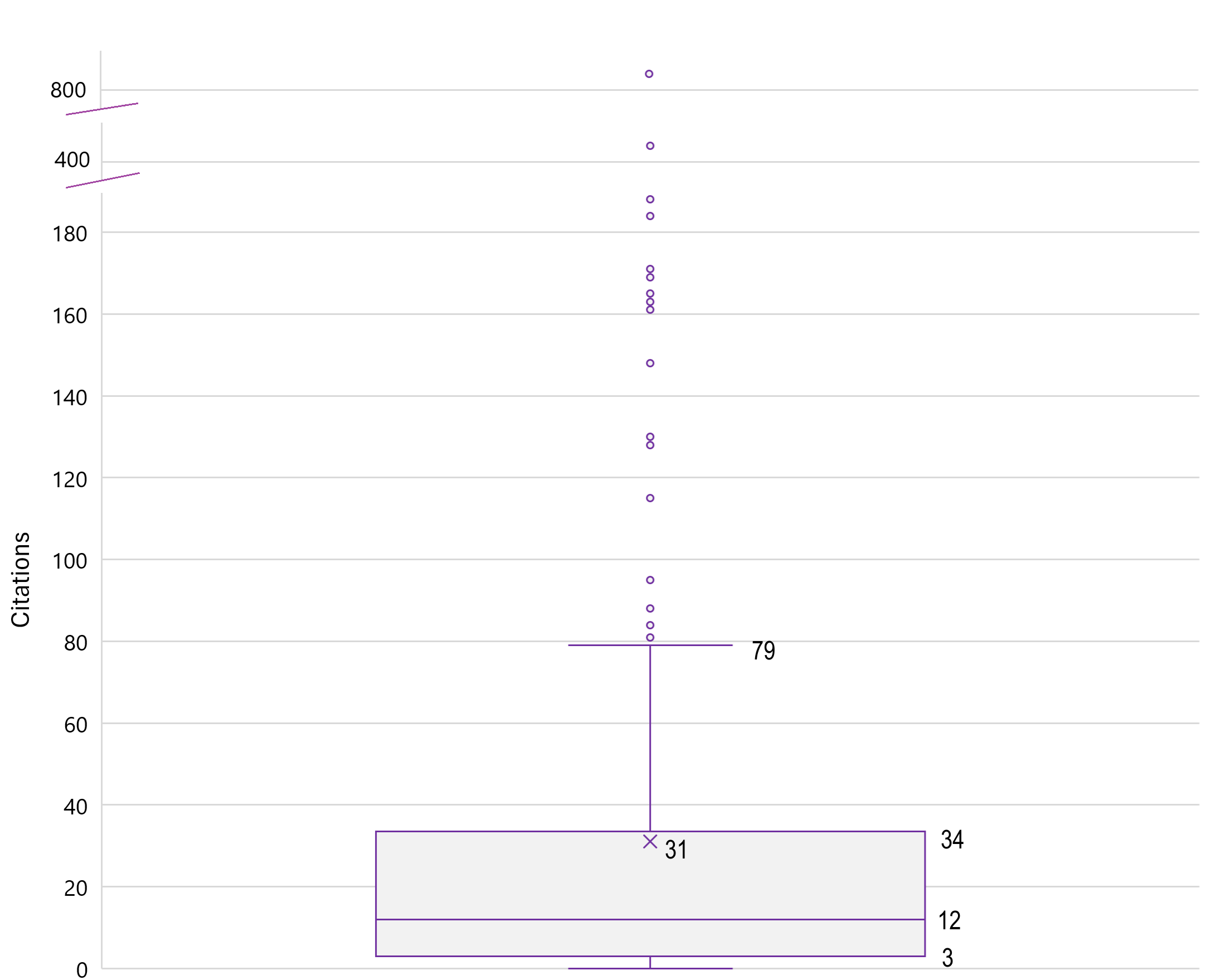

Supplement: Supplementary file 1 [file sports-13-00145-s001.zip › Figure S2. Box Plot Isometric.png]

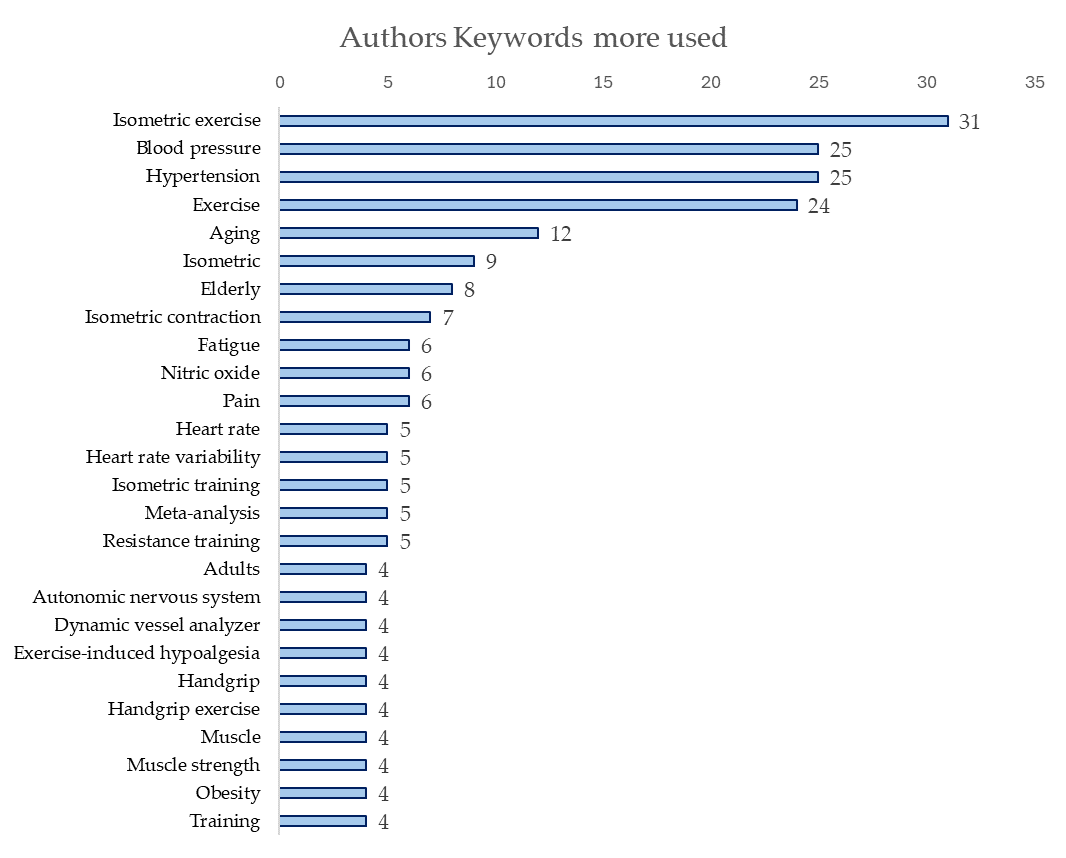

Supplement: Supplementary file 1 [file sports-13-00145-s001.zip › Figure S3. Authors Keywords.png]

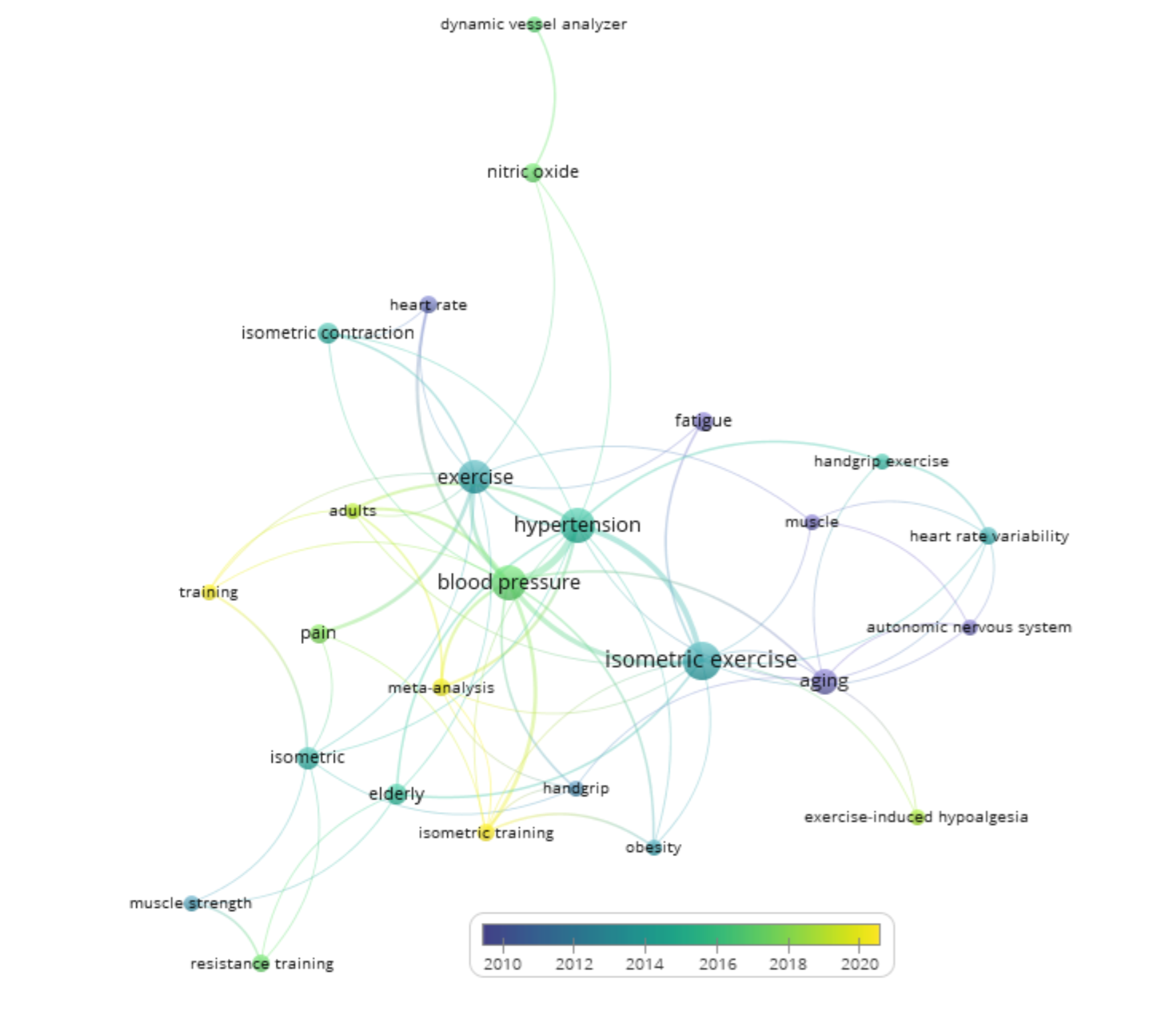

Supplement: Supplementary file 1 [file sports-13-00145-s001.zip › Figure S4 AK as 5_0 AVG PUB YEAR.png]

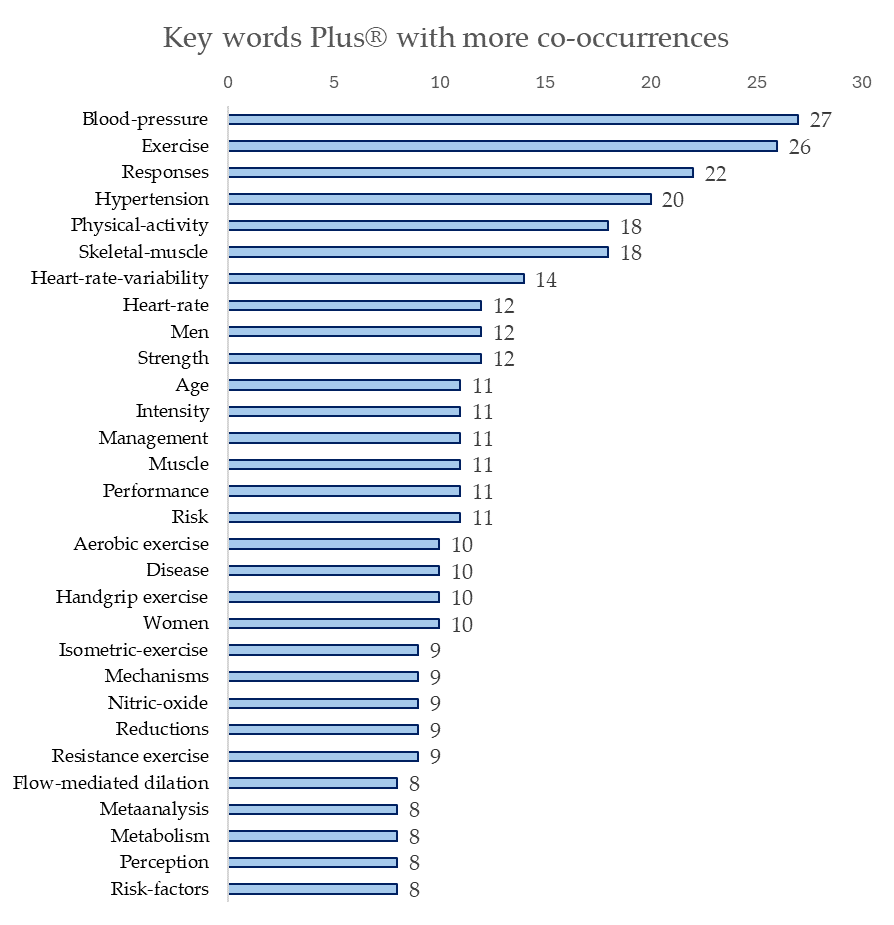

Supplement: Supplementary file 1 [file sports-13-00145-s001.zip › Figure S5 Keywords Plus.png]

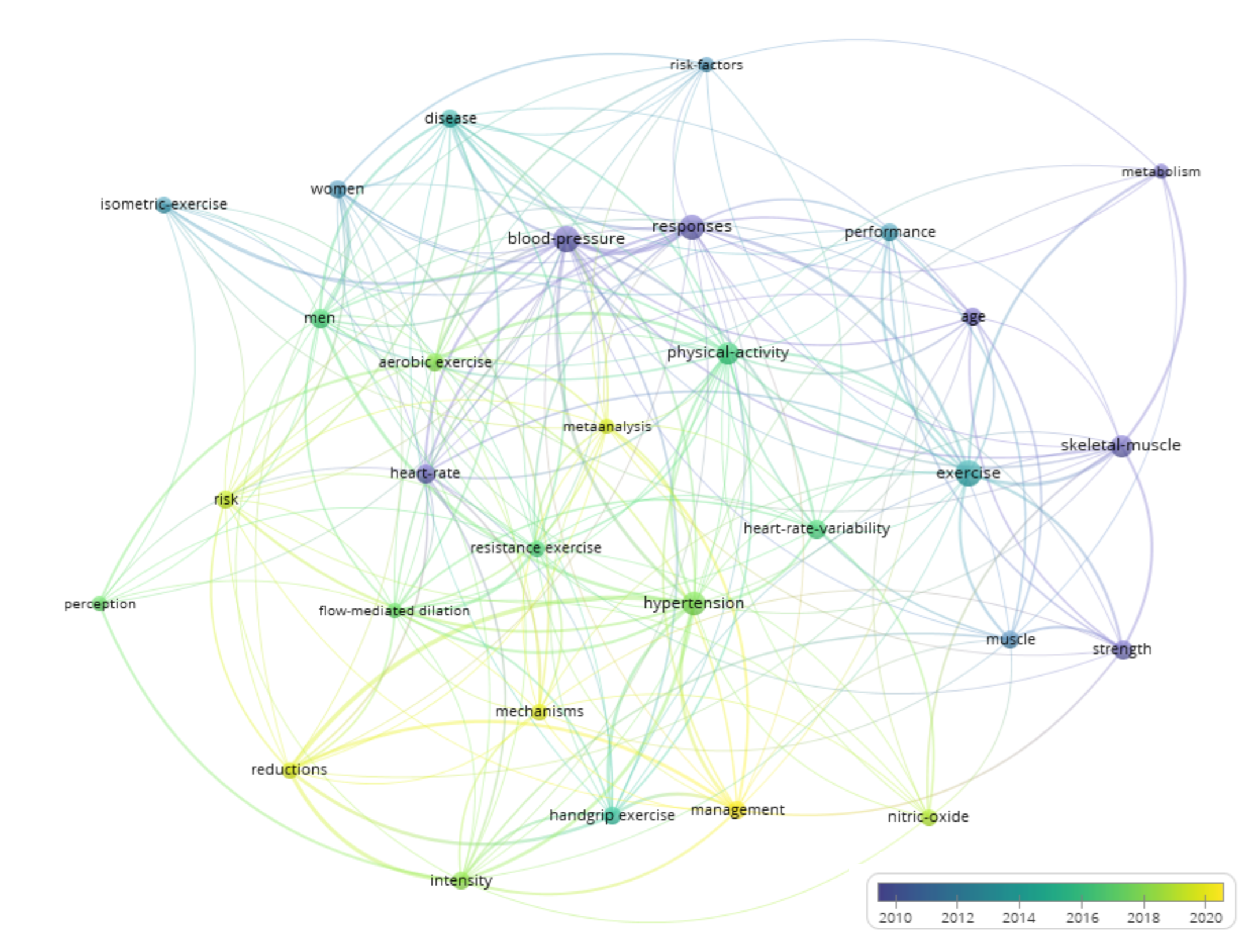

Supplement: Supplementary file 1 [file sports-13-00145-s001.zip › Figure S6 KP as 5_0 AVG PUB YEARS.png]
